# Oral health behaviour of children and adolescents in Germany. Results of the cross-sectional KiGGS Wave 2 study and trends

**DOI:** 10.17886/RKI-GBE-2018-096

**Published:** 2018-12-12

**Authors:** Laura Krause, Benjamin Kuntz, Liane Schenk, Hildtraud Knopf

**Affiliations:** 1 Robert Koch Institute, Berlin, Department of Epidemiology and Health Monitoring; 2 Charité – Universitätsmedizin Berlin, Institute of Medical Sociology and Rehabilitation Science

**Keywords:** CHILDREN AND ADOLESCENTS, TOOTH BRUSHING FREQUENCY, DENTAL CHECK-UPS, HEALTH MONITORING, KIGGS

## Abstract

Oral health behaviour plays a key role in the prevention of caries and periodontitis. This article describes the prevalence, determinants and trends of tooth brushing frequency and utilization of dental check-ups. The analyses are based on the data from the second wave of the German Health Interview and Examination Survey for Children and Adolescents (KiGGS Wave 2, 2014-2017). The results show that around 80% of children and adolescents meet the recommended tooth brushing frequency and utilization of dental check-ups. Around one fifth of children and adolescents do not meet the recommendations. 14- to 17-year-old adolescents, as well as those with low socioeconomic status and a migration background are groups which are particularly at risk. Compared to the KiGGS baseline study (2003-2006), tooth brushing frequency and utilization of dental check-ups has improved. While this positive development is apparent for nearly all the population groups analysed, the same risk groups that were identified by the baseline study are also evident in the KiGGS Wave 2 results. Targeted measures directed at specific target groups to promote oral health behaviour at younger ages should therefore be maintained and expanded, respectively.

## 1. Introduction

Caries belongs to the most frequent diseases in childhood and adolescence [1]. The great spread of the disease can be traced back to insufficient oral hygiene and inadequate nutrition [2]. Effective oral hygiene and regular dental check-ups (see info box) are, besides adequate fluoridation and nutrition that prevents caries, pillars in the prevention of tooth and mouth diseases [3-6]. This is also reflected in the guidelines on caries prophylaxis for permanent teeth [4] as well as the guidelines of the Bundesausschuss der Zahnärzte und Krankenkassen on tooth, gum and jaw disease early detection examinations (dental check-ups according to sentence 2 of section 26 (1) of Book 5 of the German Social Code) [7].

### 1.1 Recommendations on oral health behaviour

According to recommendations, the more frequently the teeth are brushed with fluoride toothpaste the greater degree to which caries can be prevented [8, 9]. The aim is to completely remove bacterial plaque from tooth surfaces. The German Society of Dentistry and Oral Medicine (DGZK) recommends brushing milk teeth with a thin film of fluoride containing children’s toothpaste once a day. From age two, a child’s milk teeth should be brushed two times a day with a pea-sized amount of fluoride toothpaste for children. After the first permanent teeth appear, fluoride toothpaste for adults should be applied twice a day [8, 9]. As children cannot initially be expected to brush their teeth by themselves, parents are responsible for the dental hygiene of their children until approximately the age of eight. However, when children are two years old parents should begin teaching them to brush their teeth by themselves [10].

**KiGGS Wave 2**

Second follow-up to the German Health Interview and Examination Survey for Children and Adolescents

**Data owner:** Robert Koch Institute

**Aim:** Providing reliable information on health status, health-related behaviour, living conditions, protective and risk factors, and health care among children, adolescents and young adults living in Germany, with the possibility of trend and longitudinal analyses

**Study design**: Combined cross-sectional and cohort study

**Cross-sectional study in KiGGS Wave 2**

**Age range:** 0-17 years

**Population:** Children and adolescents with permanent residence in Germany

**Sampling:** Samples from official residency registries - randomly selected children and adolescents from the 167 cities and municipalities covered by the KiGGS baseline study

**Sample size:** 15,023 participants

**KiGGS cohort study in KiGGS Wave 2**

**Age range:** 10-31 years

**Sampling:** Re-invitation of everyone who took part in the KiGGS baseline study and who was willing to participate in a follow-up

**Sample size:** 10,853 participants

**KiGGS survey waves**

► KiGGS baseline study (2003-2006), examination and interview survey
► KiGGS Wave 1 (2009-2012), interview survey
► KiGGS Wave 2 (2014-2017), examination and interview survey

More information is available at

www.kiggs-studie.de/english

According to the guidelines of the Federal Joint Committee, children up to and including age five should go for dental check-ups to detect diseases of the teeth, mouth and jaw at least once a year, and 6- to 17-year-old children and adolescents at least twice [7, 11].

Group prophylaxis to improve oral health and prevent tooth, gum and jaw diseases (section 21 SGB V) is directed comprehensively at all children in day-care up to the age of 6, 6- to 12-year-old children at school, as well as 12- to 16-year old adolescents at school or facilities for persons with disabilities that present an increased risk of caries. Measures include an examination of the oral cavity, of the condition of teeth and enamel hardening, as well as target group adapted advice on nutrition and oral hygiene. Specific programmes should be developed for children and adolescents that present a particularly high risk of caries [12]. Moreover, individual prophylaxis entitles 7- to 17-year-old children and adolescents to an individual caries prophylaxis at a dentist twice per year (section 22(1) SGB V) that has a function in preventive care and diagnosis.

Beyond group and individual prophylaxis, 2.5- to 6-year-old children are legally entitled to three check-up examinations to detect tooth, gum and jaw diseases (section 26 SGB V). The first of these examinations should take place during a child’s third life year, the other two before the child’s sixth birthday [7]. These examinations aim to detect diseases and abnormal developments of the teeth, gum and jaw. They are also directed at increasing parents’ and children’s awareness of dental hygiene and tooth-friendly nutrition. On this basis, the Preventive Health Care Act (Präventionsgesetz, PrävG), which was passed in 2015, provided for further dental check-ups for infants and small children to prevent caries at early ages [13]. Since 2016, the ‘yellow booklet’ (where early detection examinations are recorded) has continuously promoted dental prevention through six legally binding referrals to a dentist for children between 6 and 64 months old [14].

### 1.2 Caries among children and adolescents in Germany

Germany’s fifth oral health study (DMS V), conducted by the Institute of German Dentists (IDZ) between 2013 and 2014, provides population representative data on oral health for 12-year-olds in Germany [15]. The data shows that dental prophylaxis and adequate dental care has led to an impressive decrease in the levels of caries prevalence among children in Germany over the last 25 years. 12-year-olds on average today have 0.5 decayed, filled or missing teeth due to caries. In 1997, the figure was still 1.7. In international comparison, Germany has a leading position [15].

In spite of this significant drop, a large proportion of children and adolescents still suffer from caries. This is particularly evident for early childhood caries of the milk teeth, where decay of the milk teeth due to caries occurs before a child’s third birthday [16-18]. In the epidemiological examinations that accompanied group prophylaxis examinations in 2016, the German Working Committee for Dental Care of Youths (DAJ) in its last study for the first time collected data on early childhood caries prevalence in Germany [19]. The dentition of 11.4% of 3-year-olds required treatment, 2.3% had already received treatment. 81.3% had naturally healthy teeth [19]. As with 12-year-olds, the prevalence of caries is increasingly polarised, with an increasingly smaller group of children and adolescents presenting the largest number of decaying teeth. Oral health studies indicate socioeconomic status (SES) as a key factor in the development of caries [19-21]. Prevalence of caries is not only significantly higher among children and adolescents from socially disadvantaged families [22, 23], but also among girls and boys with a migration background [24-26].

**Info box Oral health behaviour**

Dental care at home (such as using fluoride toothpaste to brush your teeth) and professional dental cleaning by a dentist mechanically remove bacterial plaque. During dental check-ups, the teeth and gums are checked for diseases of the teeth, mouth and jaw.

School entry examinations in Berlin too, during which doctors of public health services control the teeth and gum health of children at school entry age, reveal the role of social differences for dental health [27]. According to the data collected in 2016, the teeth of 30.8% of children with low SES either required treatment, were decayed or missing, whereas the corresponding figure for children with medium and high SES was significantly lower (9.7% and 2.5%, respectively). A similar correlation is apparent for migration background. 24.9% of children with a two-sided migration background, 9.8% of children with a one-sided migration background and 5.8% of children without a migration background had teeth that either required treatment, were decayed or missing [27].

### 1.3 Oral health behaviour of children and adolescents in Germany

DMS V data shows that roughly one in two children up to the age of 12 knows the dental care recommendations and brushes their teeth properly [15]. We can thereby ascribe the existing socioeconomic differences in dental health to differences in oral health behaviour. The proportion of children and adolescents from families with low status who know the recommendations for dental health is lower, they brush their teeth less often and do not go to dental check-ups as frequently as their peers from the high status group [15]. The representative data from the German Health Interview and Examination Survey for Children and Adolescents (KiGGS baseline study, 2003-2006) also highlights this fact, as nearly one third of children and adolescents present insufficient oral health behaviour [24, 26]. The proportion of adolescents who do not brush their teeth often enough and have fewer dental health check-up examinations was highest among those with low SES and a migration background [26].

The second follow-up survey, KiGGS Wave 2 (2014-2017), again provides representative data on oral health for 0- to 17-year-old children and adolescents in Germany. This article now describes the prevalence of tooth brushing frequency and utilization of dental check-ups differentiated according to sociodemographic factors such as age, gender, SES, migration background, size of municipality (rural/urban) and region (East and West German federal states (including Berlin)). To highlight the potential for prevention measures, the focus is on groups which are particularly at risk. A comparison with the KiGGS baseline study (2003-2006) reveals the trends during the last ten years. The article uptake of orthodontic treatment by children and adolescents in Germany in this issue of the Journal of Health Monitoring presents the initial results from KiGGS Wave 2 on the utilization of orthodontic treatment.

## 2. Methodology

### 2.1 Sample design and study conduct

KiGGS is part of the health monitoring system at the Robert Koch Institute (RKI) and includes repeated cross-sectional surveys of children and adolescents aged 0 to 17 (KiGGS cross-sectional study) that are representative for Germany. The KiGGS baseline study (2003-2006) was conducted as an examination and interview survey, the first follow-up study (KiGGS Wave 1, 2009-2012) as a telephone-based interview survey and KiGGS Wave 2 (2014-2017) as an examination and interview survey. The concept and design of KiGGS have been described in detail elsewhere [28-31]. Participants were selected randomly from the official registries of the 167 cities and municipalities representative for Germany which had already been chosen for the KiGGS baseline study. A number of activities have been conducted to increase study participation and to improve the sample composition [29, 32]. 15,023 children and adolescents (7,538 girls, 7,485 boys) took part in KiGGS Wave 2 (2014-2017) (response rate 40.1%). Response rates were calculated based on the Response Rate 2 of the American Association for Public Opinion Research (AAPOR) [6]. 17,641 girls and boys aged 0 to 17 (8,656 girls, 8,985 boys) took part in the KiGGS baseline study, with a response rate of 66.6% [30].

### 2.2 Indicators of oral health

In line with the KiGGS baseline study (2003-2006), indicators on tooth brushing frequency were measured in KiGGS Wave 2 using the self-reported information provided by the participants (11- to 17-years-old) or their legal guardians (0- to 10-years-old) on a questionnaire completed in writing. The questions asked were ‘How often are your child’s teeth brushed,’ or ‘How often does your child brush its teeth?’ or ‘How often do you brush your teeth?’ The answers were ‘at least twice a day’, ‘once a day’, ‘several times a week’, ‘once a week or less’ and ‘not at all’ and were summarised in keeping with the recommendations for tooth brushing frequency. Accordingly, 0- to 1-year-old children should brush their teeth at least once a day and 2- to 17-year-olds at least twice per day with an appropriate fluoride toothpaste (see recommendations on oral health behaviour) [8, 9]. For the group of 0- to 10-year-olds, parents or legal guardians responded on the utilization of dental check-ups, 11- to 17-year-olds provided the answers themselves. The question was: ‘How often does your child have dental checkups?’ and ‘How often do you have dental check-ups?’ The answers ‘every three months’, ‘every six months’, ‘once per year’, less than once per year’ and ‘I have never been to the dentist’ were summarised according to the recommendations [7]. For up to 5-year-old children, the recommendation is to have a dental check-up once and for 6- to 17-year olds twice a year (see recommendations on oral health behaviour) [7, 11]. Additional dental check-ups for infants and small children to prevent early childhood caries were only introduced at the point of data collection for KiGGS Wave 2 (2014-2017), meaning that this article only presents the utilization for children older than 3.

The article now focuses on the proportion of children and adolescents not meeting the recommendations for tooth brushing frequency and utilization of dental checkups (risk group).

### 2.3 Determinants of oral health behaviour

Previous studies have identified that SES in particular and migration background are determinants for childhood oral health behaviour in addition to age, gender and place of residence [24, 26, 33]. KiGGS determines SES based on the responses derived from the information provided by the parents about their education, occupational status and income (equivalised disposable income). Based on an index that applies a point sum which equally reflects all three indicators a distribution-based differentiation of three groups is established, according to which 20% of children and adolescents belong to the low (1st quintile), 60% to the medium (2nd to 4th quintile) and 20% to the high status group (5th quintile) [34]. Migration background is defined based on the child’s country of birth as well as parent country of birth and citizenship. A one-sided migration background was defined as having one parent who was not born in Germany or does not hold German citizenship. The group of two-sided migration background included children who had themselves migrated to Germany and have at least one parent who was not born in Germany, or whose parents were both born in a country other than Germany or are non-German nationals [32].

### 2.4 Statistical methods

The first part of the cross-sectional analysis is based on data from 14,121 respondents aged 0 to 17 years (7,115 girls, 7,006 boys) with valid answers on tooth brushing frequency. The second part of the cross-sectional analysis is based on the data from 12,926 respondents aged 3 to 17 years (6,493 girls, 6,433 boys) with valid answers on the utilization of dental check-up examinations. The results are presented as prevalences (frequencies) with 95% confidence intervals and are stratified according to gender, age, SES, migration background, size of municipality (rural/urban) and region (East and West German federal states (including Berlin)). Associations between tooth brushing frequency and/or utilization of dental check-ups and the selected determinants were calculated by applying multivariate logistic regression models, which produced results in the form of odds ratios. These are to be interpreted as a risk ratio and indicate the factor by which the risk of a low frequency of tooth brushing or low utilization of dental check-ups is increased in comparison to the reference group. In order to achieve reliable findings on the significance of the individual determinants on child and adolescent oral health behaviour they were mutually statistically controlled. The calculations were carried out using a weighting factor that corrects deviations within the sample from the population structure with regard to regional structure (rural area/urban area), age (in years), gender, federal state (as at 31 December 2015), German citizenship (as at 31 December 2014) and the parents’ level of education (Microcensus 2013 [35]).

The first part of the trend analysis is based on data from the KiGGS baseline study (2003-2006) collected from the 16,764 respondents aged 0 to 17 years (8,216 girls, 8,548 boys) with valid responses on tooth brushing frequency. The second part of the trend analysis is based on the data of 14,278 respondents aged 3 to 17 years (7,005 girls, 7,273 boys) with valid answers on the utilization of dental check-ups. Trends between the baseline study (2003-2006) and KiGGS Wave 2 (2014-2017) were calculated based on age and gender standardised prevalences (as at 31 December 2015) at both survey points, and the difference tested by univariate logistic regression analysis. Here, a new weighting factor was applied to the data from the baseline study that accounts for parental levels of education and federal state in line with KiGGS Wave 2 (in addition to the factors included in original weighting). An analysis of trends by SES and migration background used the modified cross-sectional weightings from the KiGGS baseline study and KiGGS Wave 2 reflecting an adaptation to official population statistics with regard to time of survey.

All analyses were conducted with Stata 15.1 (Stata Corp., College Station, TX, USA, 2015). The analyses were based on the data set of the KiGGS baseline survey (version 25) and KiGGS wave 2 (version 11). Stata survey procedures were applied in all analyses to account for the clustering of participants at examination points and the weighting in the calculation of confidence intervals and p-values in an appropriate way [36]. A statistically significant difference between groups is assumed to have been demonstrated in cases where the p-value was lower than 0.05.

## 3. Results

### 3.1 Tooth brushing frequency

According to KiGGS Wave 2 data, 77.7% of 0- to 17-year-olds meet the tooth brushing frequency recommendations. Conversely, this implies that 22.3% of children and adolescents do not brush their teeth frequently enough. Table 1 shows the risk groups among children and adolescents that do not brush their teeth as frequently as recommended. The results show that boys run a higher risk, compared to girls, of not brushing their teeth frequently enough. Moreover, this risk increases with age: compared to 0- to 1-year-old infants, 11- to 17-year old children and adolescents have nearly twice as high a risk of not brushing their teeth frequently enough. Furthermore, the results indicate a social gradient. Children and adolescents of medium and, in particular, low status groups meet the tooth brushing recommendations significantly less than their peers in the high status group. The migration background of respondents has a similar effect: children and adolescents with a two-sided migration background meet the tooth brushing frequency recommendations less often than their peers with one-sided migration background, who, in turn, meet the recommendations less often than those with no migration background. According to the data, place of residence has no effect on tooth brushing frequency.

### 3.2 Dental check-ups

KiGGS Wave 2 data shows that 80.3% of 3- to 17-year-olds have dental check-ups according to the recommendations. Conversely, this means that 19.7% of children and adolescents do not meet the recommendations. Table 2 shows risk groups of children and adolescents for a low utilization of dental check-ups. There are no differences between girls and boys. Compared to the 3- to 5-year-old reference group, 6- to 10-year-olds and 11- to 13-year olds run nearly twice as great a risk and 14- to 17-year-olds run nearly three times as great a risk of not having dental check-ups according to the recommendations. The results also highlight the role played by socioeconomic status. Compared to children and adolescents with high SES, their peers with low SES run nearly twice as high a risk of not meeting the dental check-up recommendations but children and adolescents with medium SES run a lower risk. In line with the results regarding tooth brushing frequency, the risk of low use of dental check-ups for children and adolescents with a migration background is gradually increasing. There are also differences regarding place of residence: not having dental check-ups according to the recommendations is therefore more frequent in large towns and cities than in rural areas and more frequent in the former West German than in the former East German federal states.

### 3.3 Oral health behaviour trends

**Figure 1:**
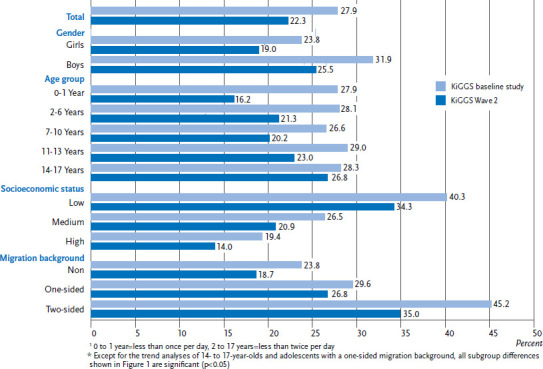
Trend of tooth brushing frequency that does not meet the recommendations^1^ among 0-to 17-year olds according to gender, age, socioeconomic status and migration background (KiGGS baseline study n=8,216 girls, n=8,548 boys; KiGGS Wave 2 n=7,115 girls, n=7,006 boys)* Source: KiGGS baseline study (2003-2006), KiGGS Wave 2 (2014-2017)

**Figure 2:**
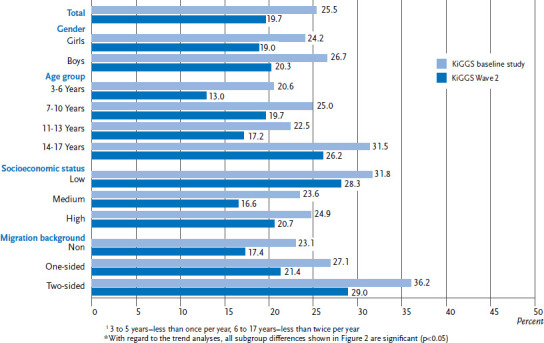
Trend of utilization of dental check-ups not meeting the recommendations^1^ among 3-to 17-year-olds according to gender, age, socioeconomic status and migration background (KiGGS baseline study n=7,005 girls, n=7,273 boys; KiGGS Wave 2 n=6,493 girls, n=6,433 boys)* Source: KiGGS baseline study (2003-2006), KiGGS Wave 2 (2014-2017)

Between the survey points of the KiGGS baseline survey (2003-2006) and KiGGS Wave 2 (2014-2017), the proportion of children and adolescents who do not brush their teeth in accordance with the recommendations decreased significantly (Figure 1). This positive development is apparent for both genders, in nearly all age groups, all status groups, as well as for children and adolescents without and with two-sided migration backgrounds. The exception is 14- to 17-year-old adolescents and adolescents with one-sided migration backgrounds. Among these groups, the proportion of those who do not brush their teeth frequently enough has remained stable at a high level. Compared to the KiGGS baseline study the proportion of children and adolescents that do not have dental check-ups according to the recommendations has also decreased significantly in KiGGS Wave 2 (Figure 2). This positive development is observed across all the population groups analysed.

## 4. Discussion

KiGGS Wave 2 data shows that 77.7% of 0- to 17-year-olds brush their teeth according to the recommendations. Accordingly at 22.3%, around one fifth of children and adolescents do not brush their teeth frequently enough. Data from the 2013/2014 study Health Behaviour in School-aged Children (HBSC) can be used for interpreting the results [33]. The study found that 20.8% of pupils do not brush their teeth frequently enough. Although this figure is nearly identical to the preva lences found in KiGGS Wave 2, a direct comparison is not possible due to differences in the age groups (0-17 and 11-15 years, respectively) and the operationalisation of tooth brushing [33]. However, national and international studies do confirm KiGGS Wave 2 data regarding the fact that there are groups of children and adolescents with a greater risk of not brushing their teeth frequently enough [15, 26, 33, 37, 38]. Among them are boys, 14- to 17-year-old adolescents, children and adolescents with low SES, as well as those with one-sided and in particular two-sided migration backgrounds.

KiGGS Wave 2 shows that the vast majority of children and adolescents, i.e. 80.3% do meet the recommendations regarding the utilization of dental check-ups. This implies that 19.7%, or nearly one fifth of 3- to 17-year-olds do not have dental check-ups regularly enough. The utilization of measures of group and individual prophylaxis can help assess the use of dental check-ups [39]. Consequently, in line with the reports of the German Working Committee for Dental Care of Youths (DAJ), measures of group prophylaxis reached 2 million children in day-care centres (77.8% of all children in daycare centres) and 2.2 million in primary school (78.5% of all children in primary school) during the 2015/2016 school year. In classes 5 and 6 it was 464,911 pupils (32.6% of all children and adolescents in secondary school classes 5 and 6), 87,396 pupils in classes 7 to 10 (3.2% of all children and adolescents in secondary school classes 7 to 10) and 177,486 of those in special schools (74.4% of all adolescents in special schools) [40]. As regards the utilization of dental check-ups, data from the statutory health insurer BARMER GEK shows that 35.1% of children had dental check-ups. 65.7% of children and adolescents made use of individual prophylaxis [41]. Overall, the data from health insurance and the DAJ reveal a great potential for increasing the utilization of dental check-ups among children and adolescents. KiGGS results allow children and adolescents living in cities and the former West German federal states to be identified as risk groups. 14- to 17-year-old adolescents, children and adolescents with low SES, as well as those with one-sided and indeed two-sided migration background are particularly at risk.

A comparison of the results on oral health behaviour from KiGGS Wave 2 (2014-2017) with the results from the KiGGS baseline survey (2003-2006) shows a significant decrease in the proportion of children and adolescents who do not meet the recommendations for tooth brushing frequency and utilization of dental checkups. This positive development is seen across almost all population groups. An exception is 14-to-17-year-old adolescents and children and adolescents with one-sided migration backgrounds, among whom the proportion of those that do not brush their teeth frequently enough has remained stable at a high level over the ten year period considered. Regarding the prevention of early childhood caries, fortunately the proportion of 6-year-olds with inadequate oral health behaviour has dropped significantly. Unlike children and adolescents in the low status group, the utilization of dental check-ups by children and adolescents with high and in particular medium SES has increased significantly between the two survey points. KiGGS data showed in particular an increase over time in the utilization for children and adolescents of the medium status group also for paediatric and gynaecological services [42, 43]. The risk groups for inadequate oral health behaviour that were identified by the KiGGS baseline study were also found in KiGGS Wave 2. Adolescents in general, as well as those with low SES and migration background deserve special attention because the rates for not meeting the recommendations on tooth brushing frequency and utilization of dental check-ups continue to be the highest for these groups.

KiGGS Wave 2 (2014-2017) data confirms the findings of the KiGGS baseline study (2003-2006) of regional differences between East and West Germany for the utilization of dental check-ups [26]. The first two German oral health studies (DMS) (1989 and 1992) highlighted differences between East and West in the oral health of children and adolescents [15]. One likely reason were the differences between the healthcare systems in the East and West. Public health care in the former German Democratic Republic (GDR) achieved good results: according to DMS II, 12-year-olds in East Germany had less carious teeth and more often a caries free dentition than 12-year-olds in West Germany. Following the reunification of Germany, the health political panorama in Germany changed profoundly (for example introduction of group and individual prophylaxis, broader use of fluoride in toothpastes). Gradually the figures for caries and caries free dentition for 12-year-olds in the former West and East German federal states converged [44] and, according to DMS V hardly differ today [15]. The fact that children and adolescents in the former West German federal states do not meet the recommendations for the utilization of dental check-ups more often than their peers in the former East German federal states could also be down to the greater number of dentists per person in the former East German federal states[39].

The KiGGS baseline study (2003-2006) for the first time provided representative data on the oral health behaviour of children and adolescents in Germany across the entire age range of 0 to 17. The results were of high public health relevance and can now be continued with KiGGS Wave 2 (2014-2017). However, the interpretation of results needs to consider that the values were self-reported by respondents. It cannot be ruled out that the results may be distorted by socially desirable response behaviour and that the proportion of children and adolescents with oral health behaviour that does not comply with the recommendations may be underestimated [45]. Nonetheless, the results of other studies on tooth brushing frequency and utilization of dental check-ups produce figures similar to those found in KiGGS Wave 2 [33, 40, 41].

### Conclusion and outlook

The population-based cross-sectional results of KiGGS Wave 2 provide up-to-date information on tooth brushing frequency and utilization of dental check-ups, as well as the factors related to oral health behaviour and facilitate an assessment of developments over time. While the oral health behaviour of children and adolescents regarding the indicators analysed has improved over the last ten years, the data nonetheless highlights further potential for prevention.

Generally, it is important to prevent caries as early as possible [46]. To achieve this requires dentists, gynaecologists and obstetricians, paediatricians and midwives to work closely together. They need to inform children and adolescents as well as their parents and motivate them to go to dental check-ups [46]. The health target ‘Health before, during and after birth’ highlights the importance of caries prevention at early childhood and in this connection emphasises providing pregnant women and future parents with information and explanations [47]. Target group appropriate measures, such as for adolescents or for children and adolescents with low socioeconomic status and migration background have delivered promising results [46]. In this regard, daycare centres and schools play a key role here as settings [37, 48].

Studies reveal that the tooth brushing behaviour acquired in early childhood remains a relatively stable pattern and is generally maintained into adult age [49]. Preserving gum and tooth health at child and adolescent age is of great importance because any damage to permanent teeth is irreversible and will permanently influence oral health in all later life phases [26]. Longitudinal analyses on the individual development of oral health behaviour across the life course from childhood, through adolescence and into young adulthood will, in future, become possible with the data respondents of the KiGGS cohort provide when they are contacted again in the future [50].

## Key statements

- Around 80% of children and adolescents fulfil the recommendations for tooth brushing frequency and utilization of dental check-ups.
- Around one fifth of children and adolescents do not brush their teeth often enough and do not meet the recommendations for dental check-ups.
- 14- to 17-year-olds as well as children and adolescents with low socioeconomic status and a migration background are risk groups for insufficient oral health behaviour.
- Compared to the baseline study, the proportion of children and adolescents with inadequate oral health behaviour has decreased in KiGGS Wave 2.

## Figures and Tables

**Table 1:** Proportion of 0- to 17-year-olds that do not meet the tooth brushing frequency recommendations^1^ according to gender, age, socioeconomic status, migration background, size of municipality and place of residence (n=7,115 girls, n=7,006 boys) Source: KiGGS Wave 2 (2014-2017)

%		(95% Cl)	OR	(95% Cl)	p-value
**Total**	22.3	(21.2-23.4)			
**Gender**					
Girls	19.0	(17.5-20.5)	Ref.		
Boys	25.5	(24.0-27.0)	**1.50**	(1.31-1.69)	< 0.001
**Age group**					
0-1 Year	16.2	(12.9-20.2)	Ref.		
2-6 Years	21.3	(19.2-23.6)	**1.44**	(1.08-1.93)	0.015
7-10 Years	20.2	(18.2-22.4)	**1.33**	(0.98-1.82)	0.068
11-13 Years	23.0	(21.0-25.3)	**1.67**	(1.23-2.28)	0.001
14-17 Years	26.8	(24.6-29.1)	**1.87**	(1.38-2.53)	< 0.001
**Socioeconomic status**					
Low	34.3	(31.4-37.4)	**2.60**	(2.15-3.16)	< 0.001
Medium	20.9	(19.6-22.4)	**1.54**	(1.63-2.36)	< 0.001
High	14.0	(12.5-15.7)	Ref.		
**Migration background**					
Non	18.7	(17.7-19.8)	Ref.		
One-sided	26.8	(23.7-30.2)	**1.62**	(1.35-1.94)	< 0.001
Two-sided	35.0	(31.6-38.5)	**1.94**	(1.61-2.32)	< 0.001
**Size of municipality**					
Rural (<5,000 inhabitants)	22.7	(20.2-25.4)	Ref.		
Small town (5,000–<20,000 inhabitants)	21.9	(20.2-23.8)	0.92	(0.77-1.10)	0.367
Middle-sized town (20,000–<100,000 inhabitants)	21.4	(19.0-24.1)	0.83	(0.68-1.01)	0.061
Large cities (≥100,000 inhabitants)	23.3	(21.2-25.4)	0.86	(0.71-1.05)	0.086
**Place of residence**					
East German federal states	19.2	(17.7-20.9)	Ref.		
West German federal states (including Berlin)	22.8	(21.5-24.1)	1.06	(0.94-1.21)	0.470

^1^ 0 to 1 year=less than once per day, 2 to 17 years=less than twice per day

CI=Confidence interval, OR=Odds Ratio, Ref.=Reference, Bold=statistically significant compared to the reference group (p<0.05)

**Table 2:** Proportion of 3- to 17-year-olds that do not meet the recommended dental check-ups^1^ according to gender, age, socioeconomic status, migration background, size of municipality and place of residence (n=6,493 girls, n=6,433 boys) Source: KiGGS Wave 2 (2014-2017)

%		(95% CI)	OR	(95% CI)	p-value
**Total**	19.7	(18.4-21.1)			
**Gender**					
Girls	19.0	(17.5-20.6)	Ref.		
Boys	20.3	(17.5-20.6)	1.07	(0.97-1.20)	0.184
**Age group**					
3-5 Years	13.0	(11.3-15.0)	Ref.		
6-10 Years	19.7	(17.6-21.9)	**1.66**	(1.34-2.05)	< 0.001
11-13 Years	17.2	(15.3-19.3)	**1.44**	(1.16-1.78)	0.001
14-17 Years	26.2	(23.8-28.8)	**2.40**	(1.99-2.88)	< 0.001
**Socioeconomic status**					
Low	28.3	(24.9-31.9)	**1.26**	(1.03-1.55)	0.024
Medium	16.6	(15.2-18.1)	**0.75**	(0.65-0.87)	< 0.001
High	20.7	(19.1-22.5)	Ref.		
**Migration background**					
Non	17.4	(16.0-18.8)	Ref.		
One-sided	21.4	(18.5-24.6)	**1.24**	(1.01-1.51)	0.035
Two-sided	29.0	(25.6-32.7)	**1.56**	(1.28-1.89)	< 0.001
**Size of municipality**					
Rural (<5,000 inhabitants)	14.9	(12.7-17.5)	Ref.		
Small town (5,000–<20,000 inhabitants)	17.1	(14.6-20.0)	1.12	(0.86-1.45)	0.413
Middle-sized town (20,000–<100,000 inhabitants)	21.0	(18.4-23.5)	**1.33**	(1.05-1.68)	0.018
Large cities (≥100,000 inhabitants)	23.6	(21.4-26.1)	**1.46**	(1.15-1.85)	0.002
**Place of residence**					
East German federal states	13.8	(12.2-15.5)	Ref.		
West German federal states (including Berlin)	20.7	(19.2-22.2)	**1.32**	(1.10-1.57)	0.003

^1^ 3 to 5 years=less than once per year, 6 to 17 years=less than twice per year

CI=Confidence interval, OR=Odds Ratio, Ref.=Reference, Bold=statistically significant compared to the reference group (p<0.5)
